# Effect of trimethoprim on *Calliphora stygia* (Diptera: Calliphoridae) larvae growth and detection via liquid chromatography–tandem mass spectrometry

**DOI:** 10.1093/jme/tjaf066

**Published:** 2025-06-20

**Authors:** Donna B McIntyre, Kalyan Pantha, Philip S Barton, Benjamin M Long

**Affiliations:** Future Regions Research Centre, Federation University, Mount Helen, VIC, Australia; Graduate Research School, Federation University, Mount Helen, VIC, Australia; Future Regions Research Centre, Federation University, Mount Helen, VIC, Australia; Graduate Research School, Federation University, Mount Helen, VIC, Australia; School of Life and Environmental Sciences, Deakin University, Geelong, VIC, Australia; Future Regions Research Centre, Federation University, Mount Helen, VIC, Australia

**Keywords:** entomotoxicology, *Calliphora stygia*, liquid chromatography–tandem mass spectrometry, post-mortem interval

## Abstract

Insects are crucial in the estimation of time-since-death of remains due to their predictable growth. Pharmaceuticals present in decomposing remains may alter insect growth and could affect postmortem interval calculations. Here we report on an investigation into the effects of trimethoprim, on the growth of *Calliphora stygia* (Fabricius, 1781) (Diptera; Calliphoridae) larvae, a common species of blow fly in Australia. We asked: (i) does trimethoprim influence larval growth, and (ii) can trimethoprim be detected in *C. stygia* larvae reared on trimethoprim? We found a significant increase in larval length in the trimethoprim-exposed group relative to the control group as accumulated degree hours increased. By mid-late experiment (~3,000 accumulative degree hours), larvae exposed to trimethoprim measured significantly longer than the control larvae; 15 to 18 mm (SD = 2.5 mm), representing a 164% increase compared to the control, which measured 11 to 13 mm (SD = 3.0 mm). We also observed a significant effect of trimethoprim on larval instar development and larval mass, however the latter was accompanied by higher variability. Additionally, trimethoprim was detected in all larvae (analyzed via HPLC–MS/MS) reared on antibiotic-treated substrates, indicating choice of growth models can be important when using larval length to estimate postmortem interval. Our findings highlight the potential for trimethoprim to affect larval growth of *C. stygia*, and likely other blow flies. This has implications for interpreting growth models used in postmortem interval estimations, and the need to expand knowledge of entomotoxicology into forensic investigations where data on the effects of pharmaceuticals on local insect species remains limited.

## Introduction

The decomposition of human and animal remains is complex and highly variable (Benbow et al. 2019). This poses a challenge for accurately determining the minimum postmortem interval (PMI_min_) of deceased individuals (Dawson et al. 2023). One method used to assess PMI_min_ is the analysis of insect evidence (typically blow flies) by retrospectively calculating the age of key species of larvae using established growth curves. Yet different insect species exhibit varying development rates and require specific optimal conditions for growth (Amendt et al. 2004). Therefore, it is essential to study these larval growth rates under a wide range of controlled environments to establish reliable references for entomology-based PMI_min_ estimations.

Various chemicals can affect the development rates of insects, and therefore estimation of PMI_min_, but research in this area has largely been limited to drugs of dependence (McGrath and Jenkins 2009, Desrosiers and Watterson 2010, AbouZied 2016, Al-Khalifa et al. 2021, Al-Qahtni et al. 2021), or household products (Charabidze et al. 2009, Schotsmans et al. 2014, Aubernon et al. 2015, McIntyre et al. 2022). These chemicals may alter the natural rates of development of larvae due to interference with cellular or other biological processes.

Various studies have explored the impact of antibiotics on insect physiology and development, yielding mixed results. Antibiotics can reduce the activities of antioxidant enzymes, and cause oxidative damage to silkworm intestine, as confirmed by histopathological analysis (Li et al. 2020). Antibiotics may not affect the palatability of food for insects, but can alter the diversity of both food and intestinal microorganisms in *Hermetia illucens* (Linnaeus 1758) (Diptera; Stratiomyidae), inhibiting its growth and development (Zhang et al. 2022). By contrast, no differences in larval survival, maturation rate, or pupal weight were observed in *Lucilia sericata* (Meigen 1826) (Diptera; Calliphoridae) reared on media with the antibiotics ampicillin, ceftizoxime, and vancomycin, (Sherman et al. 1995). A study on trimethoprim found it slowed larval development in *Pimpla turionellae* (Linnaeus 1758) (Hymenoptera: Ichneumonidae), with the negative effects mitigated by adulthood; though this study did not assess changes in larval length and weight (Büyükgüzel 2002). Faster growth was observed in *Spodoptera litura* (Fabricus 1775) (Lepidoptera: Noctuidae) larvae fed diets supplemented with various concentrations of streptomycin sulfate (0.03%, 0.07%, and 0.15%), compared to diets without it (Thakur et al. 2016). The streptomycin-supplemented diet led to increased digestive enzyme activity and decreased detoxifying enzyme activity, alongside shifts in gut microbial diversity.

Global antibiotic use has increased by 46% since 2000 (Browne et al. 2021), highlighting the importance of understanding how antibiotics may impact the development of larvae in species relevant to forensic investigations (Tomberlin et al. 2017, Rawat et al. 2023). The effects of antibiotics on insect growth is complex and varies depending on the type of antibiotic and insect species investigated. Therefore, we selected trimethoprim, a widely prescribed antibiotic (Nibamureke 2019), to investigate its impact on the growth of the golden-haired blow fly, *Calliphora stygia* (Fabricus, 1781) (Diptera; Calliphoridae), a common and forensically significant species in south eastern Australia (Kavazos and Wallman 2012), which is a primary colonizer of remains, and oviposits around body orifices or wounds (Parry et al. 2011).

We asked 2 questions in this study: does trimethoprim effect growth of *C. stygia* larvae, as measured by length, mass and instar? And; can trimethoprim be detected in *C. stygia* larvae reared on trimethoprim?

Results from our study could have implications for the interpretation of growth models of blow flies exposed to antibiotics and improve entomotoxicological knowledge of a forensically important blow fly.

## Methodology

We conducted our study using 6 45 × 30 cm ventilated containers, each covered with mesh to prevent larval suffocation or escape. Three replicate containers served as negative controls, while the remaining 3 replicates were designated for the trimethoprim treatment. All trial replicates were conducted simultaneously. The treatment containers were supplied with trimethoprim at a concentration of 9.6 μg g^−1^ of kangaroo meat. This was the calculated to be the approximate equivalent to the muscle concentration in a single dose of trimethoprim in an adult (200 mg/65 to 75 kg) every 12 h with a half-life of 8 to 10 h (Brogden et al. 1982, AA PHARMA 2014, Fryar et al. 2021). Each container housed 150 larvae. Kangaroo meat used throughout all laboratory experiments was purchased at a supermarket in Ballarat, Australia. Kangaroo meat was chosen as a substrate as it was cheap, readily available, and lacked the enzymes present in beef liver (typical substrate) responsible for biological pharmaceutical degradation. *Calliphora stygia* larvae were purchased from a bait shop in Geelong, Australia and reared to adulthood in an insect dorm.

### Larval Set Up and Sampling

The colony was protein-starved for 6 d prior to the experiment. Kangaroo meat was then warmed to ambient temperature (approximately 25 °C) before feeding the colony. The next day, 50 g of kangaroo meat was provided to collect any precocious larvae for 30 min, after which it was replaced with another container of kangaroo meat (approximately 150 g) for another 2 to 3 h to allow sufficient egg-laying (approximately 2,000 to 3,000 eggs). This was then transferred to another plastic container with mesh and labeled with the time the eggs were collected. Once the eggs had hatched (approximately 24 h after being laid), they were counted for the experiment. Larvae were reared in 25 °C, 37% humidity, and exposed to 12:12 photoperiod.

After the first instar larvae had hatched, they were transferred to a piece of kangaroo meat using tweezers. The pieces of meat, each carrying 150 larvae, were then transferred to each container with 2.5 g of meat per larva (375 g total) placed in the containers.

Prior to the placement of the larvae, the meat in the treatment containers were hand mixed with trimethoprim at a concentration of 9.6 μg g^−1^ of kangaroo meat for 5 min to ensure even distribution (George et al. 2009). Control group meat was used as is. This was the calculated to be the approximate equivalent to the muscle concentration in a single dose of trimethoprim in an adult. After placement of the larvae, wet paper towels were placed on top of the meat (control and treatment) and were sprayed with tap water every 12 h to prevent desiccation. The laboratory was within a controlled temperature environment of 25 °C, and temperature data was converted to accumulative degree hours (ADH) by summating the temperature every hour. This data was then used as a temporal scale of time.

Eight larvae were randomly sampled from each container at 12-h timepoints and hot water killed by immersing in off the boil water (>80 °C) for 30 s (Amendt et al. 2011) before analysis. Nearing the end of the experiment ~3,000 ADH, sampling timepoints were reduced as there were lower larval densities. Larvae were not sampled once wandering occurred. The larvae were then measured for length and instar under a dissection microscope (with graduated ruler) and weighed on a calibrated weight scale to 0.0000 mg accuracy (Ohaus, Melbourne, Adventurer AX224 model). The developmental stage of each larvae was identified based on the number of separated spiracular slits present in each posterior spiracle (Gennard 2012). A ruler measuring to 1 mm was placed under the microscope to allow for measuring of the larvae. Four of the larvae were then placed in ethanol, with the other 4 placed in −20 °C freezer until they were freeze dried for HPLC–MS/MS analysis.

### High Performance Liquid Chromatography–Tandem Mass Spectrometry

For the HPLC analysis, freeze-dried larvae were ground into a fine powder and placed in 1.5 ml tubes. Each sample was spiked with 10 μl of caffeine as an internal standard (1 µg ml^−1^), while the positive control samples were spiked with 10 μl of caffeine (1 µg ml^−1^) and 10 μl of trimethoprim (1 µg ml^−1^). Methanol (1 ml) was added to both the control and sample tubes. The mixtures were then vortexed for 1 min, sonicated for 15 min, and centrifuged at 3,500 rpm for 15 min. This extraction process was repeated 2 additional times, and the 3 methanol extracts were combined, yielding a total of 3 ml of extract. The pooled extracts were frozen at −20 °C for 1 h to precipitate fats, then filtered through 0.45 μm regenerated cellulose syringe filters. The filtered extracts were evaporated to dryness under a nitrogen gas flow and reconstituted with 1 ml of Milli-Q water. Finally, the solution was filtered through 0.45 μm filters directly into HPLC vials for further analysis.

The analysis of trimethoprim in *C. stygia* larvae was performed using a Shimadzu LCMS-8030 equipped with a Kinetex C18 column. A gradient elution was employed, increasing the acetonitrile content from 3% to 100% over 12.51 min while maintaining a constant 0.1% formic acid concentration. The mobile phase was held at 100% acetonitrile for an additional 4 min. Following this, the column was re-equilibrated for 5 min before the next injection. A needle wash solution containing a 1:1 mixture of methanol and Milli-Q water, followed by pure Milli-Q water, was used. The mass spectrometer operating parameters were as follows: nebulizing gas flow 3 liter min^−1^, heating block temperature 400 °C, DL temperature 250 °C, and drying gas flow 15 liter min^−1^.

### Multiple Reaction Monitoring Optimization

The multiple reaction monitoring (MRM) mode was optimized for the detection of the trimethoprim using 100 ng ml^−1^ standard. A table of MRM conditions is provided in the Supplementary Information (Table S1).

### Pharmaceutical Standard and Quantification

Trimethoprim was obtained locally from Federation University, Ballarat, in sealed container. The sample was dissolved in Milli-Q water. Five point calibration standards were prepared by serial dilution to cover a range of 0.1, 0.5, 1, 5, and 10 ng ml^−1^ and measured multiple times (*n* = 8). Calibration data were shown to be in the linear range and was modeled using a weighted linear regression using 1/concentration as the weighting factor (*R*^2^ = 0.9954, *P* < 0.001, Figs. S1 and S2). Heteroscedasticity was tested for using a nonconstant variance test (*χ*^2^ = 2.360824, df = 1, *P* = 0.124), variable independence was tested for using the Durbin–Watson Statistic (Statistic = 1.66, *P* = 0.274). After quantification of trimethoprim as ng ml^−1^ it were converted to ng g^−1^ based on individual maggot masses.

### QA/QC

Throughout the procedure, instrument and method blanks were analyzed, and standards were rerun to ensure there were no carry over events and that analysis were stable. Method blanks consisted of the larvae from the control group. Instrument blanks consisted of both Milli-Q water injections and nil injections. The limits of detection (LOD) and quantification (LOQ) were calculated based on the standard deviation of the response and the slope as LOD = 0.0488 ng ml^−1^ and LOQ = 0.148 ng ml^−1^. In addition, all chromatograms were checked to comply with a 10:1 signal-to-noise ratio. Spike recoveries were conducted using larvae from the control group with 77.7 ± 0.6% trimethoprim (*n* = 3) recovered at the 10 ng ml^−1^ level as a measure of method accuracy and precision. Mass spectrometry chromatograms of a method blank and spike recovery are provided in the Supplementary Information (Fig. S3).

### Data Analysis

All analyses were conducted using the R base package version 2023.12.0 + 369 (Team 2010) while plots were created using the ggplot2 package (Wickham and Wickham 2016), dcr and ggpubr (Ritz et al. 2015, Kassambara 2018).

### Model Fitting and Evaluation

For larval instar, mean length and mean mass, we fitted sigmoidal models by ADH, using the LL.2, LL.4, and LL.3 function from the drc package, respectively. Best model fit was chosen after investigating the best fit of several models and analyzing ANOVA and sum of squares. To assess the quality of the models, we plotted residuals vs. fitted values to check for any patterns in the residuals. In addition to the formal test, we visually inspected the residuals using *Q–Q* plots to assess if they followed a normal distribution. Although the LL.4 fit showed a lower sum of squares for the larval mass, we chose the LL.3 fit as it was not a statistically significant difference between the fits, as it was for the length data. Full statistical data analysis can be observed in Tables S2 to S4 in the Supplementary Materials.

### Homogeneity of Variances

We performed Levene’s test to check if the variances were equal and examine the variability between the treatment groups (Control and Trimethoprim).

For testing larval length and weight differences between treatments, we conducted Welch’s ANOVA with adhoc pairwise *t-*tests with a Bonferroni correction.

To assess the variability in the mean mass of larvae, we calculated the coefficient of variation (CV) for each group.

To test the differences between larval instar development between the groups, at differing timepoints of ADH, we employed Mann–Whitney *U* tests. We used a 2-parameter logistic model to determine the ADH at which 50% of larvae developed from second to third instar, then employed a *t*-test to determine any significant differences between treatment groups.

## Results

**Fig. 1.**
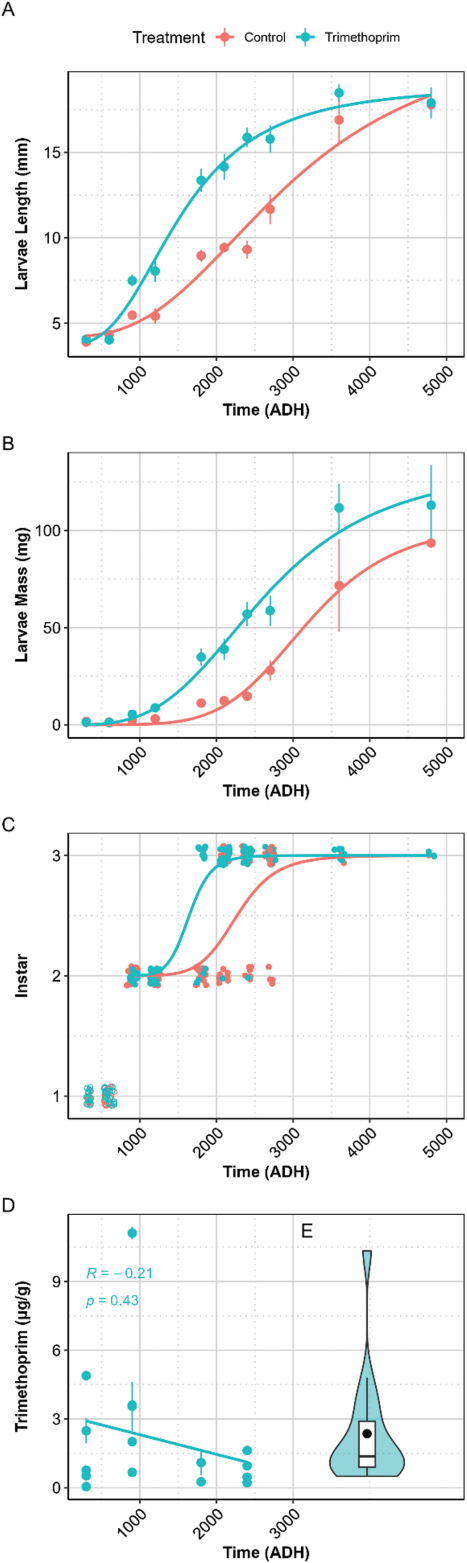
A) Mean larvae length (mm), B) mean larvae mass (mg), C) larval development between instars, and D) concentration of trimethoprim detected in larval samples (µg g^−1^) with standard error bars over time as ADH.

Larvae length varied (*P* < 0.001, df = 1.00, *F*(1.00, 235.00) = 33.41) between the control and trimethoprim larvae length over time measured ADH (Fig. 1). The larvae began with the same length at the start of the experiment, however, as ADH increased, those in the trimethoprim containers grew significantly larger throughout the experiment. By mid-late experiment (~3,000 ADH), the average length of the larvae exposed to trimethoprim has already reached 15 to 18 mm, compared to 11 to 13 mm in the control group. Similarly, larvae mass varied (*P* < 0.001, df *=* 1.00, *F*(1.00, 188.00) = 36.73) over time (Fig. 1) as the experiment progressed. However, a large CV (50 ± 4%) highlighted substantial variability in mass as a measure vs length as a measure of growth (18 ± 2%).

The significant interaction (*P* ≤ 0.002, df = 1.00, *F*[1.00, 308.00] = 28.00 [length], 98.00 [weight]) between treatment and ADH, indicated that the effect of trimethoprim was dependent on time (ADH). Specifically, the treatment had a lower significant effect on larval growth at the beginning of the experiment but differentiated between groups as ADH increased and continued until the final 2 sampling timepoints, whereby on average, the trimethoprim-treated larvae reached maximum length more rapidly than the control larvae. The final replicates of trimethoprim and control larvae wandered from their food source to pupate at the same time, despite a significant difference in instar developmental rates at 1,800 (*P* = < 0.001, *W* = 38), 2,100 (*P* = 0.002, *W* = 61.00), and 2,400 (*P* = 0.04, *W* = 96.50) ADH. As can be observed in Fig. 1, a significant difference was observed at the ADH corresponding to the transition of 50% of larvae from the second to the third instar between the treatments (*P* < 0.001, *Z* = −3.31). There were no significant differences observed or able to be calculated at other timepoints due to lack of variability in the data.

Trimethoprim was detected by HPLC–MS/MS (Fig. S4) in all analyzed samples reared in the trimethoprim groups, with varying concentrations (Fig. 1), and a mean concentration of 2.14 (± 0.7) µg g^−1^. With the exception of an outlier, as larvae mass increased, the concentration of trimethoprim (μg) per mg of larvae decreased over time as ADH. The concentration of trimethoprim over time displayed a slightly negative correlation which was insignificant.

## Discussion

To our knowledge, this is the first investigation into the impact of the antibiotic trimethoprim on insect growth as measured by effects on larval length and mass, with instar previously reported by Büyükgüzel (2002). Our results show that trimethoprim can alter the developmental trajectory of *C. stygia.* This potentially leads to inaccurate PMI_min_ estimation, particularly when using ADH-based models in forensic entomology. The larger size of the larvae in the presence of trimethoprim could lead to an overestimation of the PMI if forensic investigators rely solely on larval development data without considering the presence of antibiotics such as trimethoprim. For example, a specimen of ~15 mm size exposed to trimethoprim could be interpreted to be up to 2,700 ADH, which is an overestimation of 300 ADH (equivalent to 12 h at 25 °C). Larvae exposed to trimethoprim reached 50% of their growth (length) by 1,540 ADH, compared to those in the control group which was 2,960 ADH. This is a difference of 1,420 ADH, or 56 h (more than 2 d), which is substantial in a forensic investigation. This finding aligns with previous research in entomotoxicology, which has demonstrated that various drugs and toxins can alter insect development and, consequently, PMI estimations (Goff and Lord 1994, Carvalho et al. 2001, AbouZied 2016, Ishak et al. 2018).

Despite significant differences in instar developmental rates at 1,800, 2,100 and 2,400 ADH, the final replicates of both trimethoprim-exposed and control larvae exhibited synchronized wandering behavior and pupation. This suggests that while trimethoprim exposure influenced earlier developmental stages, it did not ultimately affect the timing of pupation. The absence of significant differences at other time points was due to a lack of variability in the data. These findings indicate that trimethoprim may accelerate certain instar transitions but does not necessarily alter the overall duration of larval development leading to pupation, a finding also reported by Büyükgüzel (2002).

There are several plausible mechanisms for antibiotics to effect growth of larvae, and it is important to note that this is a complex process in which both beneficial and pathogenic microbes in larvae may be reduced and/or affected. As such, we speculate that the significantly larger size of larvae exposed to trimethoprim may be attributed to the antibiotic’s disturbance of the local microbiome, both beneficial and pathogenic (Dillon and Dillon 2004, Valzania et al. 2018, Preußer et al. 2021, Zhao et al. 2022). It is possible that the trimethoprim ingested by the larvae reduced the presence of harmful bacteria and inhibited bacterial reproduction, thereby enhancing larval growth. Supporting this hypothesis, Zhao et al. (2022) found that insects exposed to antibiotics exhibited reduced gut microbiome diversity, which in turn benefited host growth, development, and reproduction. Our findings, however, contrast with those of Zhang et al. (2022), who found metronidazole and levofloxacin inhibited insect growth at low doses in *H. illucens*. This discrepancy may be explained by differences in the insect species examined, the internal flora of the species, as well as the distinct mechanisms of action of the antibiotics used.

Our mass data showed differences between the control and trimethoprim-treated larvae, but substantial variability in mass within each timepoint suggests a more complex relationship between antibiotic exposure and larval growth, as supported by the CV value. The variability may be due to factors such as differential absorption of the antibiotic, genetic variation among the larvae, or other environmental conditions that were not controlled in this study (Lee et al. 2022). This variability not only complicates the interpretation of mass data but also suggests that mass may not be a reliable metric for assessing larval growth in the context of forensic entomology and as such, do not recommend it.

The delayed and time-dependent effects of trimethoprim on larval growth highlight the need to account for drug exposure when estimating the PMI_min_. Early growth may appear unaffected, but significant impacts emerge over time, potentially skewing PMI estimates in later stages. The plateau of effects suggests larvae may adapt or reach a threshold where the drug no longer inhibits growth. These findings demonstrate the importance of considering cumulative drug effects and developmental timing in forensic entomology, particularly in toxicological analysis.

Our research detected trimethoprim in all analyzed samples of larvae reared on trimethoprim-treated substrates at a μg/g level. The ability of larvae to acquire trimethoprim from their food source has important implications for forensic entomotoxicology, as it confirms that insect larvae can serve as reliable indicators of chemical substances present in the environment or within decomposing remains. This information is particularly valuable when conventional toxicological samples, such as blood or tissue, are unavailable or degraded (Bugelli et al. 2017). The presence of trimethoprim also raises important questions regarding how different pharmaceuticals, beyond just illicit drugs, can influence insect development and potentially confound PMI estimates.

Given the rising global use of antibiotics, it is increasingly likely that forensic investigations will encounter remains that have been exposed to such substances. Without accounting for the impact of antibiotics like trimethoprim, PMI estimations could be skewed, potentially affecting the outcomes of criminal investigations as PMI estimations are used to provide a timeline of events (Introna et al. 2011, Cockle and Bell 2015, Benedetti et al. 2019, Mansour 2019). Another important consideration to the effect of trimethoprim on insect growth rate is there the broader implication of antibiotics in the environment, particularly in agricultural settings where antibiotics are frequently used (Islam et al. 2024). When antibiotics enter ecosystems—through runoff from livestock facilities, manure application, or improper disposal (Zhang et al. 2015, Khmaissa et al. 2024, Ugoeze et al. 2024) —they can impact nontarget organisms, including insects (Zhang et al. 2024). If exposure to antibiotics like trimethoprim or streptomycin results in faster growth rates or larger body sizes in certain insect species such as *C. stygia* and *S. litura*, this could indeed affect their fecundity, leading to potential shifts in population dynamics and consequences (eg infestations and increased incidences of fly strike [Yan et al. 2024]; or food source scarcity for higher order predators). These broader environmental and ecological ramifications compound our need to understand the effects of antibiotic exposure on insect populations. Our results also emphasize the importance of integrating entomotoxicology into forensic practice, particularly in regions like Australia, where there is limited existing data on the effects of common pharmaceuticals on local insect species.

We acknowledge that our experimental design, which involved removing larvae at 12-h intervals, may have led to decreasing larval densities over time. This could have had potential effects on both the larval development and the microbial composition of the rearing substrate.

Most blow fly larvae are known to feed communally, generating heat and digestive secretions that help suppress microbial overgrowth and facilitate nutrient breakdown (Rivers et al. 2011). A reduction in larval density over time could have impacted these communal effects, potentially altering both the nutritional quality of the substrate and the microbial environment. This, in turn, may have influenced larval growth rates, especially in later developmental stages when densities were lower.

However, we note that both the control and trimethoprim-treated groups experienced the same pattern of larval removal, meaning that any density-related effects would likely have influenced both groups similarly. If decreasing density did play a role in our findings, we would expect it to affect all treatments rather than being a confounding factor unique to trimethoprim exposure. Future studies could investigate this by maintaining a consistent larval density throughout the experiment, either by replenishing larvae or using staggered setups to better isolate the effects of trimethoprim from potential density-related influences.

This study aimed to establish proof-of-concept that an antibiotic-laced food source could be detected in larvae and to assess whether larval growth rates were affected. In a real-world scenario, compounds ingested antemortem can vary in concentration across organs and tissues due to metabolic processes. Further research is needed to explore dose–response relationships in individuals and the detectability of such compounds in insects.

## Conclusion

Our study provides a critical first step in understanding how trimethoprim affects the development of *C. stygia* larvae and its possible implications for PMI_min_ estimations. The significant differences in larval growth observed in this study suggest that forensic entomologists must consider the potential presence of antibiotics when analyzing insect evidence and that the larvae themselves may act as noninvasive indicators of antibiotics. However, the variability in mass data accentuates the need for caution when using mass as a metric for larval development. Future research should aim to explore the effects of other commonly used antibiotics on larval development, as well as investigate the underlying mechanisms driving these changes. By expanding our knowledge in this area, forensic scientists can improve the accuracy of PMI estimations and enhance the overall reliability of forensic entomology as a tool in criminal investigations.

## Supplementary material

Supplementary material is available at *Journal of Medical Entomology* online.

tjaf066_suppl_Supplementary_Tables_S1-S4_Figures_S1-S4
