# Supplementary material for: Effect of trimethoprim on Calliphora stygia (Diptera: Calliphoridae) larvae growth and detection via liquid chromatography–tandem mass spectrometry
Source: J Med Entomol. 2025 Jun 20;62(4):782–8. doi: 10.1093/jme/tjaf066 (PMC12271731; doi:10.1093/jme/tjaf066)
Supplement: tjaf066_suppl_Supplementary_Tables_S1-S4_Figures_S1-S4 [file tjaf066_suppl_supplementary_tables_s1-s4_figures_s1-s4.docx]

**Supplementary Information**

1. **Methods, page 9:** Figure S1: Calibration Curve for trimethoprim with the fitted linear model and confidence bands. Computed calibration model is “Response = 7614.5 × Concentration + 289.5”
2. Figure S2: A goodness of fit plots for Trimethoprim standards: Residual Error vs Fitted values.
3. Figure S3: Selection ion Chromatogram for the detection of Trimethoprim in Negative control (Control group larvae), Positive Control (Trimethoprim spiked control group larvae) and a 10 ng.ml^-1^ calibration standard [(*E*+) *m/z* 291.10>230.05]. Chromatograms offset for clarity (0.2 mins, 500 ion counts).
4. Figure S4: Selection ion Chromatogram for the detection of Trimethoprim [(E+) m/z 291.10>230.05]. Larvae Sample collected at 300 ADH from the Trimethoprim Group. Signal to noise ratio = 18.
5. **Methods, page 8:** Table S1: multiple reaction monitoring parameters table.
6. **Methods, page 9:** Table S2. Analysis of model of best fit for larval length and mass data
7. **Methods, page 9:** Table S3. Parameter estimates for best fit models for larval length and mass
8. **Methods, page 9:** Table S4. Parameter estimates for best fit models for larval instar.


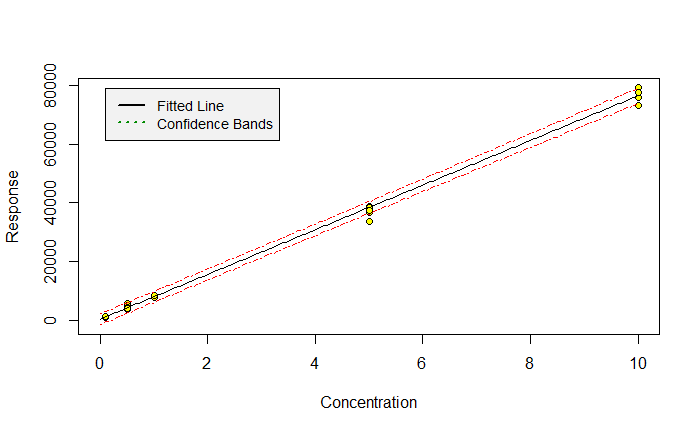


Figure S1: Calibration Curve for trimethoprim with the fitted linear model and confidence bands. Computed calibration model is “Response = 7614.5 × Concentration + 289.5”

**Alt text:** A Calibration curve for Trimethoprim


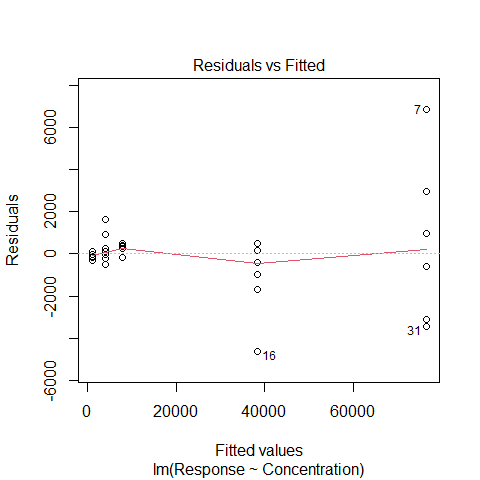


Figure S2: A goodness of fit plots for Trimethoprim standards: Residual Error vs Fitted values.

**Alt text:** A residual plot for a linear model of concentration vs detector response for trimethoprim.


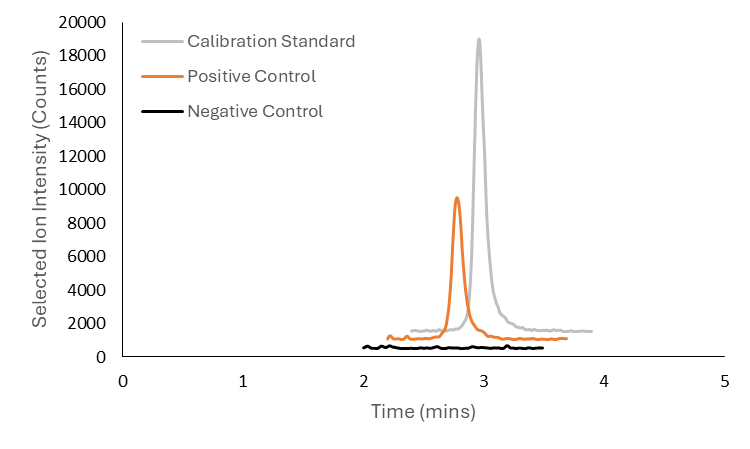


Figure S3: Selection ion Chromatogram for the detection of Trimethoprim in Negative control (Control group larvae), Positive Control (Trimethoprim spiked control group larvae) and a 10 ng.ml^-1^ calibration standard [(*E*+) *m/z* 291.10>230.05]. Chromatograms offset for clarity (0.2 mins, 500 ion counts).

**Alt text:** A chromatogram for trimethoprim in a negative control, positive control and a standard.


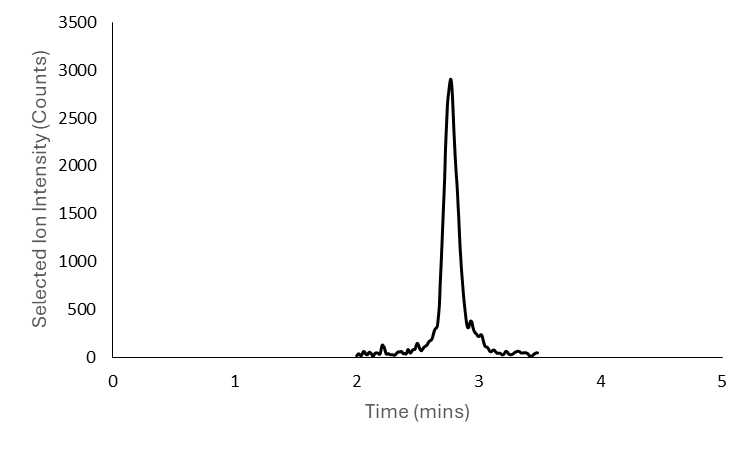


Figure S4: Selection ion Chromatogram for the detection of Trimethoprim [(*E*+) *m/z* 291.10>230.05]. Larvae Sample collected at 300 ADH from the Trimethoprim Group. Signal to noise ratio = 18.

**Alt text:** A chromatogram for trimethoprim in a larvae from the treatment group..

Table S1: multiple reaction monitoring parameters table.

| **Compound** | **Precursor Ion** | **Product Ion** | **Role** | **Dwell Time (ms)** | **Q1 Prebias (V)** | **Collision Energy (V)** | **Q3 Prebias (V)** |
| --- | --- | --- | --- | --- | --- | --- | --- |
| Trimethoprim | 291.1 | 230.05 | Quant | 100 | -12 | -28 | -21 |
|  |  | 123.10 | Qual | 100 | -16 | -30 | -27 |
|  |  | 261.20 | Qual | 100 | -12 | -33 | -18 |

Quant = Ion for quantification, Qual = ion for compound identification.

**Alt text:** A table displaying fragment parameters for multiple reaction monitoring of trimethoprim.

| **Interaction** | **Group** | **Model Type** | **Model RSE** | **Model DF** | **Model RSS** | **p (anova)** |
| --- | --- | --- | --- | --- | --- | --- |
| Length vs ADH | Control | **Four-parameter log-logistic function** | **0.996** | **6** | **5.952** | 0.0479 |
|  |  | Three-parameter log-logistic function | 1.312 | 7 | 12.042 |  |
|  |  | Linear | 1.065 | 8 | 9.069 | 0.8464 |
|  |  | Quadratic | 1.135 | 7 | 9.017 |  |
|  | Trimethoprim | **Four-parameter log-logistic function** | **0.774** | **6** | **3.598** | 0.0171 |
|  |  | Three-parameter log-logistic function | 1.195 | 7 | 10.002 |  |
|  |  | Linear | 2.395 | 8 | 45.876 | 0.0002 |
|  |  | Quadratic | 0.898 | 7 | 5.649 |  |
| Mass vs ADH | Control | Four-parameter log-logistic function | 0.00300 | 6 | 0.0000526 | 0.0749 |
|  |  | **Three-parameter log-logistic function** | **0.00365** | **7** | **0.0000931** |  |
|  |  | Linear | 0.01243 | 8 | 0.0012359 | 0.0084 |
|  |  | Quadratic | 0.00783 | 7 | 0.0004291 |  |
|  | Trimethoprim | Four-parameter log-logistic function | 0.00754 | 6 | 0.0003408 | 0.6841 |
|  |  | **Three-parameter log-logistic function** | **0.00708** | **7** | **0.0003512** |  |
|  |  | Linear | 0.01059 | 8 | 0.0008979 | 0.8490 |
|  |  | Quadratic | 0.01129 | 7 | 0.0008929 |  |

Table S2. Analysis of model of best fit for larval length and mass data

**Alt text:** Table displaying statistical analysis for each analysis of best fit for control and trimethoprim treatments, for larval length and mass versus ADH.

Table S3. Parameter estimates for best fit models for larval length and mass

| **Measure** | Length of Larvae | | | |  | Mass of Larvae | | | |
| --- | --- | --- | --- | --- | --- | --- | --- | --- | --- |
| **Model** | Four-parameter log-logistic function | | | |  | Three-parameter log-logistic function | | | |
| **Treatment** | Control | | Trimethoprim | |  | Control | | Trimethoprim | |
| **Parameter** | Estimate | p-value | Estimate | p-value |  | Estimate | p-value | Estimate | p-value |
| a | -2.7 ±1 | 0.040 | -2.8 ±0.5 | 0.0018 |  | -5.4 ±0.8 | 0.00028 | -3.4 ±0.7 | 0.0021 |
| b | 4.2 ±0.8 | 0.0021 | 3.7 ±0.8 | 0.0028 |  | 0* | - | 0* | - |
| c | 22 ±4 | 0.0032 | 19 ±0.9 | 9.9e-07 |  | 0.10 ±0.008 | 3.7e-06 | 0.13 ±0.02 | 5.9e-05 |
| d | 3000± 600 | 0.0033 | 1500 ±100 | 1.1e-05 |  | 3200 ±100 | 8.7e-08 | 2700 ±200 | 1.2e-05 |
|  |  |  |  |  |  |  |  |  |  |

* Three-parameter log-logistic function assumes a baseline of 0 as such b is not calculated.

**Alt text:** Table displaying parameter estimates for bit fit models for larval length and mass.

Table S4. Parameter estimates for best fit models for larval instar.

| Larvae Instar | | | |
| --- | --- | --- | --- |
| Two-parameter log-logistic function | | | |
| Control | | Trimethoprim | |
| Estimate | p-value | Estimate | p-value |
| -52.4 ±0.3 | <2e-16 | -41.8 ±0.4 | < 2.2e-16 |
| 2* | - | 2* | - |
| 3* | - | 3* | - |
| 734 ±1 | <2e-16 | 734 ±1 | 1.2e-05 |

**Alt text:** Table displaying parameter estimates for bit fit models for larval instar.
